# Assessment of the efficacy of 0.1% cyclosporine A cationic emulsion in the treatment of dry eye disease during COVID-19 pandemic

**DOI:** 10.22336/rjo.2023.28

**Published:** 2023

**Authors:** Wiktor Stopyra

**Affiliations:** * MW med Eye Centre, Cracow, Poland

**Keywords:** cyclosporine A, cationic emulsion, dry eye disease, ocular surface disease index

## Abstract

**Objective:** To assess the efficacy of 0.1% cyclosporine A (CsA) cationic emulsion (CE) in the treatment of dry eye disease (DED) in terms of ocular surface disease index (OSDI).

**Methods:** DED patients with corneal fluorescein staining grade (CFS) ≤ 3 on the Oxford scale and Schirmer test score < 10 mm/ 5 min were enrolled for once-daily CsA use in this observational, prospective, one-center study. Efficacy of CE at 30, 60, and 90-day follow-up visit was evaluated using OSDI questionnaire. Both the overall OSDI score and the outcomes for all subscales - ocular symptoms (OS), vision-related function (VRF) and environmental triggers (ET) were considered.

**Results:** Twelve patients (10 women and 2 men), whose baseline OSDI ranged between 27.08 and 70.03 mm (48.2 ± 11.8), were included. Their achieved mean scores for subscales such OS, VRF and ET were 66.6 ± 16.8, 42.2 ± 12.0 and 42.2 ± 12.5, respectively. Statistically significant results were obtained after 30 days for OSDI (45.5 ± 10.0; p=0.011), whereas after 90 days for both OSDI (35.4 ± 7.4; p=0.003) and OS (47.2 ± 10.9; p=0.005), VRF (30.5 ± 6.1; p=0.003) and ET (33.3 ± 11.2; p=0.008).

**Conclusions:** CsA CE significantly reduced symptoms of patients with DED. Recovery was the most successful after 90 days of treatment and included OSDI, OS, VRF and ET.

**Abbreviations:** CE = cationic emulsion, CFS = corneal fluorescein staining, CsA = cyclosporine A, DED = dry eye disease, ET = environmental triggers, OS = ocular symptoms, OSDI = ocular surface disease index, VRF = vision-related function

## Introduction

Report of the Tear Film and Ocular Surface Society International Dry Eye Workshop II concluded that DED is a multifactorial disease of the ocular surface, characterized by a loss of homeostasis of the tear film, and accompanied by ocular symptoms, in which tear film instability and hyperosmolarity, ocular surface inflammation and damage, and neurosensory abnormalities play etiological roles [**1**,**2**]. DED affects from 5 up to even 50% of the population [**3**]. DED patients complain not only of pain and sore eyes, which are sensitive to light, but also of difficulties performing basic activities of daily living, such as using a computer and driving, which are caused by visual problems such as blurry vision [**4**,**5**]. Without treatment, DED can progress, becoming more resistant to medicaments and potentially leading to permanent ocular damage [**2**,**6**].

Current treatment strategies for DED have largely been restricted to use various artificial tear formulations, which provide only short-term relief from DED symptoms and do not address ocular surface inflammation, which is the main pathophysiological component of DED [**7**,**8**]. Topical steroids have been shown to be effective in reducing the symptoms and signs of DED, however, their ocular side-effects, such as intraocular hypertension and cataract, exclude them form long-term therapy [**9**]. So, it has become obvious that other anti-inflammatory agents should be used in the treatment of DED. CsA has shown significant benefits in moderate to severe DED and has been the focus of investigation in recent years [**2**,**7**,**9**,**10**-**12**]. CsA is a lipophilic drug, so only its CE may prolong pre-corneal residence time, increase its bioavailability at the ocular surface, as well as improve corneal penetration [**9**,**13**,**14**].

The research methodology in DED is most often based on CFS, on the Oxford scale, Schirmer test score without anesthesia, tear breakup time, rarely corneal/ conjunctival lissamine green staining score on van Bijsterveld scale [**7**,**9**,**13**,**15**-**17**]. Only a few authors have considered the OSDI to estimate the efficacy of the treatment [**2**,**9**], though DEWS II report recommends the use of the OSDI (Allergan Inc, California, USA) for the assessment of the symptoms of DED [**18**]. The OSDI is an outcome of popular questionnaire that evaluates both symptom frequency and health-related quality of life [**19**]. It contains 12 questions divided into three subscales: OS - questions 1-3, VRF - questions 4-9 and ET - questions 10-12, shown in **Table 1** [**19**]. 

**Table 1:** Ocular surface disease questionnaire

Ocular surface disease questionnaire						
Have you experienced any of the following during the last week?		None	Some	Half	Most	All
1. Eyes that are sensitive to light?		□	□	□	□	□
2. Eyes that feel gritty?		□	□	□	□	□
3. Painful or sore eyes?		□	□	□	□	□
4. Blurred vision?		□	□	□	□	□
5. Poor vision?		□	□	□	□	□
Have problems with your eyes limited you in performing any of the following during the last week?	N/A	None	Some	Half	Most	All
6. Reading?	□	□	□	□	□	□
7. Driving at night?	□	□	□	□	□	□
8. Working with a computer?	□	□	□	□	□	□
9. Watching TV?	□	□	□	□	□	□
Have your eyes felt uncomfortable in any of the following situations during the last week?	N/A	None	Some	Half	Most	All
10. Windy conditions?	□	□	□	□	□	□
11. Places with low humidity (very dry)?	□	□	□	□	□	□
12. Areas that are air conditioned?	□	□	□	□	□	□

The advantages of OSDI include its translation and validation in many languages [**20**,**21**], as well as the possibility of its implementation without patient observation, which was especially valuable during the COVID-19 pandemic.

This study using the OSDI aimed to assess the efficacy of 0.1% CsA CE in the treatment of DED, in terms related to the frequency of symptoms and health-related quality of life of the patient. 

## Material and methods

The study enrolled adult patients with DED determined by CFS grade ≤ 3 on Oxford scale [**22**], a Schirmer test score without anesthesia < 10 mm/ 5 min. [**23**], as well as an OSDI score ≥ 23 [**9**], and was carried out between December 2019 and February 2021. Rigorous exclusion criteria were applied, such BCVA less than 0.5, the history of eye surgery, as well as other eye diseases.

The study was conducted adhering to the tenets of the Declaration of Helsinki. Each patient signed an informed consent for routine pharmacological treatment. 

All the patients administered 1 drop of unpreserved single-dose 0.1% cyclosporine A cationic emulsion once daily at bedtime. Each of them filled out an OSDI questionnaire four times, i.e. at baseline, on the thirtieth, the sixtieth and the ninetieth day of treatment. 

Then, the total OSDI score was calculated using the following formula:

OSDI= sum of severity for all questions answered x 100total # of questions answered x 4

where the severity was graded on a scale of 0 = none of the time, 1 = some of the time, 2 = half of the time, 3 = most of the time, 4 = all the time.

Additionally, subscales scores were computed as it follows:

OS= sum of severity for questions from 1 to 3 answered x 100total # of questions answered x 4

VRF= sum of severity for questions from 4 to 9 answered x 100total # of questions answered x 4

ET= sum of severity for questions from 10 to 12 answered x 100total # of questions answered x 4

Statistical analysis was performed using the Statistica 13.1 package. P-value < 0.05 was considered statistically significant unless it was necessary to apply Bonferroni corrections for multiple comparisons that reduced the significance level down to even 0.017. The Friedman ANOVA test was used to check statistically significant differences in the severity of the symptoms/ signs between consecutively completed questionnaires. Wilcoxon signed rank test was used to determine significant differences between specific questionnaires, i.e. 0 versus 30, 0 versus 60, and 0 versus 90. 

## Results

Twelve adult patients (10 women - 83.3% and 2 men - 16.7%) in the age range between 49 and 75 (mean 63.5 ± 7.3), were included in the study. The baseline OSDI of the studied eyes ranged between 27.1 and 70.0 (48.2 ± 11.8).

**Fig. 1:**
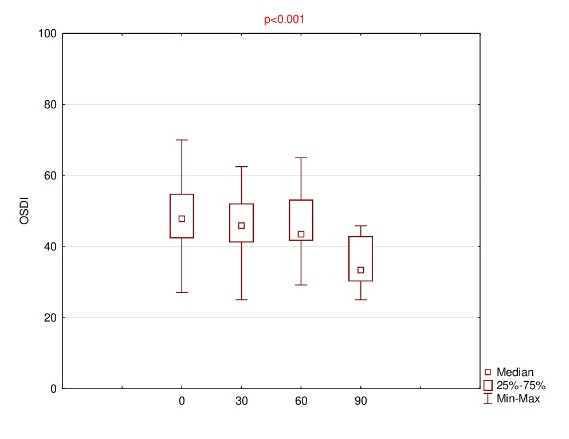
Results of OSDI for baseline (0) and follow-up (30th, 60th, 90th day of the treatment)

**Fig. 2:**
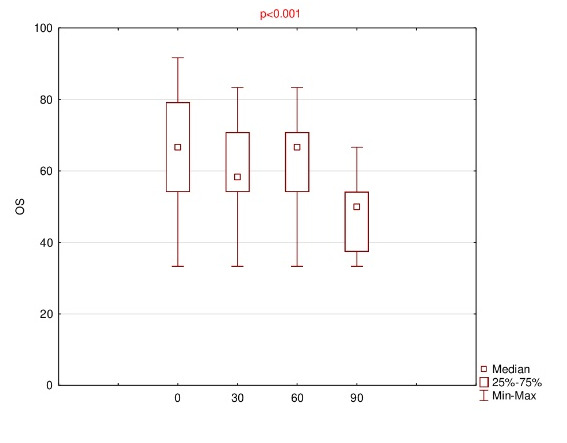
Results of ocular symptoms (OS) for baseline (0) and follow-up (30th, 60th, 90th day of the treatment)

**Fig. 3:**
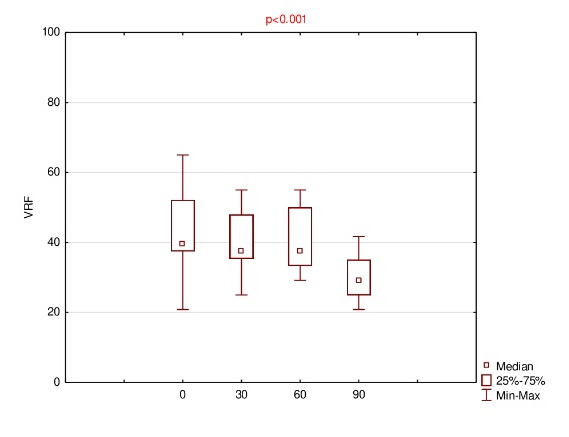
Results of vision-related function (VRF) for baseline (0) and follow-up (30th, 60th, 90th day of the treatment)

**Fig. 4:**
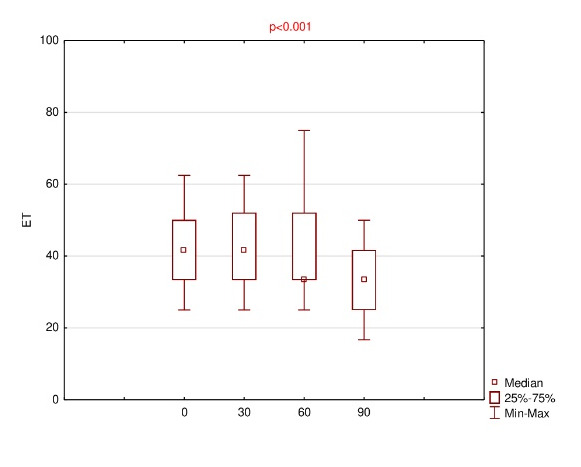
Results of environmental triggers (ET) for baseline (0) and follow-up (30th, 60th, 90th day of the treatment)

Detailed results of OSDI, OS, VRF and ET for baseline and follow-up visits were summarized using descriptive statistics - mean, standard deviation (SD), median, range and listed in **Fig. 1**-**4**.

The mean OSDI score reduced on subsequent visit up to 35.4 ± 7.4 at the end. The results of the mean of all subscales were finally decreased; however, for OS, the mean 60 > mean 30 (61.8 ± 16.5 and 61.1 ± 13.5, respectively). Anyway, using ANOVA Friedman test, it was realized that p<0.001, which was lower than the significance level both for OSDI and all subscales, e.g. OS, VRF and ET.

Therefore, using Wilcoxon signed rank test, statistically significant differences were observed for variables pairs 0 versus 30 (p=0.011) and 0 versus 90 (p=0.003) in the case of OSDI, and for pairs 0 versus 90 in the case of each of the subscales, i.e. OS, VRF and ET (p= 0.005, 0.003 and 0.008, respectively).

## Discussion

DED is one of the most common ophthalmic diseases with a great impact on the quality of life [**3**-**5**,**7**,**9**,**16**]. Although we have many drugs to treat DED, we are still looking for new substances that will improve the comfort of DED patients [**2**,**7**,**9**,**11**,**24**].

This study demonstrated that 0.1% CsA CE significantly decreased OSDI score after 90 days of the treatment (from median of 47.8 to 33.3). Moreover, a statistical reduction also applies to the results of all the subscales, i.e. OS, VRF, ET (from median of 66.7 to 50; 39.6 to 29.2; 41.7 to 33.3; respectively).

Miller et al. agreed that the overall OSDI score defined the ocular surface as normal (0-12 points), or as having mild (13-22 points), moderate (23-32 points) or severe (33-100 points) DED. In their multicenter study involving 310 patients, they proved that the minimal clinically important difference ranged from 7.0 to 9.9 for all OSDI categories, while from 4.5 to 7.3 for mild or moderate and from 7.3 to 13.4 for severe DED [**19**]. The baseline OSDI of all the patients in this study was more than 27 (moderate or severe DED) and the difference of OSDI, median after 90 days of 0.1% CsA CE treatment, was 14.4, which was more than minimal clinically important.

The SANSIKA study, conducted in 50 centers in 9 European countries as 6-month, randomized, double-masked, vehicle-controlled research, accepted patients with at least 30% improvement of OSDI as responders to the treatment of DED with 0.1% CsA CE [**7**]. Similar criteria used by Baudouin et al. in their SANSIKA’s open-label 6-month extension involved 177 patients [**9**]. In this study, 30.33% reduction of OSDI was achieved after 3 months of treatment with 0.1% CsA CE, which proved its efficacy. 

In this study, a statistically significant mild improvement of OSDI (47.8 versus 45.8) was obtained after 30 days. It was related to anti-inflammatory properties of CsA [**16**,**25**]. In the SANSIKA study, Leonardi et al. observed a significant decrease in human leucocyte antigen DR (HLA-DR) expression after only 1 month of CsA treatment [**7**]. The effect of CsA on the reduction of HLA-DR expression was observed in the SICCANOVE study, which was conducted in 61 sites located in 6 European countries involving 492 patients [**16**].

There were several limitations to the study. Firstly, relatively few patients were enrolled in the study. However, research on the efficacy of CsA in the treatment of DED in the Scottish University Teaching Hospital also involved a small number of patients [**26**]. Concomitant use of artificial tears was permitted, which might have confounded the interpretation of CsA CE effect. However, artificial tears were allowed even in the SANSIKA study [**7**,**9**]. Additionally, OSDI was the only tool to assess the efficacy of CsA CE. But, in their study, Schiffman et al. involved 139 patients and proved that OSDI demonstrated good sensitivity and specificity in the evaluation of severity of DED [**27**]. However, it is worth mentioning that the study lasted 16 months to avoid the biases from seasonal and weather-related conditions [**28**]. 

## Conclusions

The study showed that the CsA CE has a good efficacy in the treatment of DED. It reduces the patients’ symptoms and improves their quality of life. Additionally, the study proved that the OSDI is a simply but efficacious tool to assess the force of the cure of DED. Although the reliability of the presented results could be limited due to a small number of the studied group, the whole concept of such method, especially during the COVID-19 pandemic, when the contact with the patient was restricted, seemed promising.

**Conflict of Interest statement**

The author declares no conflict of interest.

**Informed Consent and Human and Animal Rights statement**

Informed consent has been obtained from all individuals included in this study.

**Authorization for the use of human subjects**

Ethical approval: The research related to human use complies with all the relevant national regulations, institutional policies, is in accordance with the tenets of the Helsinki Declaration, and has been approved by the Ethical Committee of MW med Eye Centre, Cracow, Poland.

**Acknowledgements**

None.

**Sources of Funding**

None.

**Disclosures**

None.
